# Plasma vascular endothelial growth factor levels are a potential therapy-response biomarker for pancreatic cancer

**DOI:** 10.3389/fonc.2025.1672385

**Published:** 2026-01-09

**Authors:** Christine S. Hughes, Oleg Blyuss, Hemant M. Kocher

**Affiliations:** 1 Centre for Tumor Biology, Queen Mary University of London, London, United Kingdom; 2 Barts Pancreas Tissue Bank, Barts Cancer Institute – a Cancer Research UK (CRUK) Centre of Excellence, Queen Mary University of London, London, United Kingdom; 3 Centre for Cancer Screening, Prevention and Early Diagnosis, Wolfson Institute of Population Health, Queen Mary University of London, London, United Kingdom; 4 Department of Pediatrics and Pediatric Infectious Diseases, Institute of Child’s Health, Sechenov University, Moscow, Russia; 5 Barts and the London Hepato-Pancreato-Biliary (HPB) Centre, The Royal London Hospital, Barts Health National Health Service (NHS) Trust, London, United Kingdom

**Keywords:** cytokine - immunological terms, chemokine, growth factor (VEGF), pancreatic cancer, chemotherapy, response

## Abstract

**Introduction:**

Cytokines have long been studied for their role in the pathophysiology of cancer, though their role is varied and complex. Cytokines have been mainly developed as a diagnostic or prognostic biomarker using a single measurement from a cohort of patients. Dynamic changes in cytokines may inform us about the prognostic impact of therapy under investigation.

**Methods:**

We investigated retrospectively whether a panel of selected cytokines could be used as a potential biomarker to assess treatment response and predict the prognosis of pancreatic ductal adenocarcinoma (PDAC) patients (n=19) treated with combination of Gemcitabine, nab-Paclitaxel and all-trans-retinoic acid (ATRA), in the Phase I trial (STARPAC, NCT03307148). We measured cytokine levels in the plasma samples, from multiple cycle/visit time-points.

**Results:**

Of the six cytokines (IFN-γ, IL-8, IL-16, VEGF, IL-1RA and RANTES) assessed from the STARPAC trial, we propose that VEGF could serve as a potential biomarker for eventual therapy response, as early as the second chemotherapy cycle (of six).

**Discussion:**

VEGF as a potential therapy response biomarker will need to be tested in phase II randomized controlled trial.

## Introduction

Cytokines have long been studied for their role in cancer, though their involvement is varied and complex. While in some contexts, a specific cytokine may have a tumor-suppressive effect, in others it may be tumor-promoting (1). In PDAC, there are numerous cell types found in the tumor microenvironment that can secrete and respond to cytokines; including tumor, fibroblast, stellate, endothelial, endocrine and immune cells (2–5). The subsequent cross-talk, involving multiple cytokines that may have dual, opposing effects, can be highly challenging to study, when elucidating the pathophysiology of cancer.

In this study, we assessed plasma from patients who participated in the STARPAC clinical trial. This Phase Ib trial involved treating patients with 6 cycles of a Gemcitabine - nab-Paclitaxel - ATRA combination therapy in patients with pancreatic cancer (6). Previously, we investigated serum PTX3, as a potential stromal-response biomarker. Upon ATRA treatment, serum PTX3 levels were upregulated, but this was not demonstrable by cycle 6, which suggested that perhaps ATRA treatment should not exceed 6 months (6). We also explored two ATRA-transport proteins, FABP5 and CRABP2, in the baseline biopsies of STARPAC patients, in both stromal and cancer cell compartments. Patients with increased stromal expression of FABP5 were more likely to reach disease control. Therefore, stromal FABP5 expression could be explored as potential predictive biomarker (6) and furthermore, the ratio of FABP5:CRABP2 may have a correlation with tumor progression and overall survival (7).

Having explored the stromal and cancer cell aspects, we decided to study the immune response to ATRA by focusing on the following circulating cytokines at each cycle of treatment: IFN-γ, IL-8, IL-16, VEGF, IL-1RA and RANTES (8, 9). We hypothesized that if a dynamic change in cytokine level may have a potential as a predictive or a prognostic biomarker, then the cytokine levels will be significantly different between progressed and non-progressed patients as evaluated by response evaluation criteria in solid tumors [RECIST] v1.1 criteria, as specified before (6). We also assessed dynamic CA19–9 levels and known prognostic markers such as modified Glasgow Prognostic Scale (mGPS) (10).

## Materials and methods

### STARPAC clinical trial and ethical approval

The STARPAC trial tested the combination of ATRA with two chemotherapy drugs; Gemcitabine and Nab-Paclitaxel in patients with locally advanced or metastatic pancreatic cancer (6). Patient inclusion and exclusion criteria and clinicopathological characteristics have been published with the main trial data (6). Patients received ATRA, Gemcitabine and nab-Paclitaxel in 28 day cycles. ATRA was administered for 6 cycles whereas Gemcitabine/nab-Paclitaxel were administered until disease progression and patients were followed up for 12 months. ATRA was administered orally on D1–15 of each 28 day cycle, while Gemcitabine/nab-Paclitaxel were given on D1, 8 and 15 of each 28 day cycle. The plasma samples used in this study, were collected both pre- and 5hr post-ATRA dosing on Day 1, 8 and 15 of Cycle 1, and Day 1 of subsequent Cycles (C2-C6). Ethics approval for the use of human STARPAC samples was obtained from STARPAC trial (South Central-Berkshire Research Ethics Committee (REC); 15/SC/0548) and Barts Pancreas Tissue Bank (Hampshire B Research Ethics Committee 13/SC/0592 and 18/SC/0630 and 23/SC/0324).

### Sample storage and traceability

All samples had a valid chain of custody throughout procurement, temporary storage at site, shipping, and permanent storage at the Barts Pancreas Tissue Bank (BPTB, REC Ref: 13/SC/0592, HTA License number: 12199), and were given to laboratory staff via a traceable database, in a blinded, anonymized manner.

### Plasma cytokine quantification

Plasma samples of each patient time-point were thawed only once ensuring sample stability and assayed in duplicate to facilitate reproducibility. Cytokine levels were quantified using Meso Scale Discovery V-PLEX Multiplex panels: Custom Human Biomarkers – Human IFN-γ, IL-8, IL-16, VEGF, IL-1RA (Cat. K151A9H-2) and R-PLEX Singleplex Human RANTES (Cat. F21ZN-3) assays and reported as absolute concentrations. These are sandwich immunoassays which work by applying a voltage to the plate electrodes to cause the captured labels (from labelled antibodies) to emit light. This provides a measure of the analyte present. The V-PLEX Multiplex panels were derived from Proinflammatory Panel 1, Cytokine Panel 1, Cytokine Panel 2, and as such, they all shared a similar protocol. The protocol for the R-PLEX Singleplex (RANTES) assay was slightly different, as described below.

The validated protocol used was supplied by the manufacturer. In brief, Multiplex panel plates were washed 3 times with 150uL per well of wash buffer (Cat. R61AA-1). 50uL of prepared samples or calibrators (recombinant proteins) were added per well, all in duplicates (Samples were diluted 2-fold for Proinflammatory panel 1 and Cytokine panel 1; 4-fold for Cytokine panel 2; 50-fold for R-Plex RANTES). Plates were incubated (2hrs with shaking, RT), washed 3 times with 150uL per well, and then 25uL of detection antibody solution was added to each well. Plates were incubated again (2hrs with shaking, RT), washed 3 times, and 150uL of 2x Read Buffer was added to each well. The plates were analyzed on an MSD Instrument (MESO QuickPlex SQ 120, Cat. R31QQ-3).

The Singleplex RANTES protocol differed slightly: MSD GOLD Small Spot Streptavidin plates (Cat. L45SA-2) were coated with 25uL of biotinylated capture antibody solution in each well. Plates were incubated (1hr with shaking, RT), washed 3 times, and 50uL of detection antibody solution was added to each well. Plates were incubated again (1hr with shaking, RT), washed 3 times, and then 150uL MSD GOLD Read Buffer (Cat. R92TG-4) was added per well. Diluents 7 (Cat. R54BB-3) & 8 (Cat. R54BA-3) had to be purchased separately for Singlex RANTES assay. All diluents required for Multiplex panels were provided with the kits.

Log regression graphs were constructed for standard curves. Assays were conducted in a blinded manner, and inter-day variability standard patient samples were used with acceptable coefficient of variation (<5%). Patient variables were unblinded after submission of readouts.

### Statistical analysis

Summary data are expressed as the median with interquartile range as box and whisker (min–max) plots, since the distribution was non-Gaussian. Inflammatory cytokine levels in progressed (n=9) vs. non-progressed (n=10) patients, were compared using the Mann-Whitney test for quantitative parameters (cytokines and CA199) and Fisher’s exact test for categorical (mGPS) (R version 3.5.1). All available data were included in the present analysis and missing samples (patient exit from trial, patient declining, samples unsuitable) were not imputed due to small sample size. Each data point in all presented plots corresponds to an individual patient.

## Results

**Figure 1:**
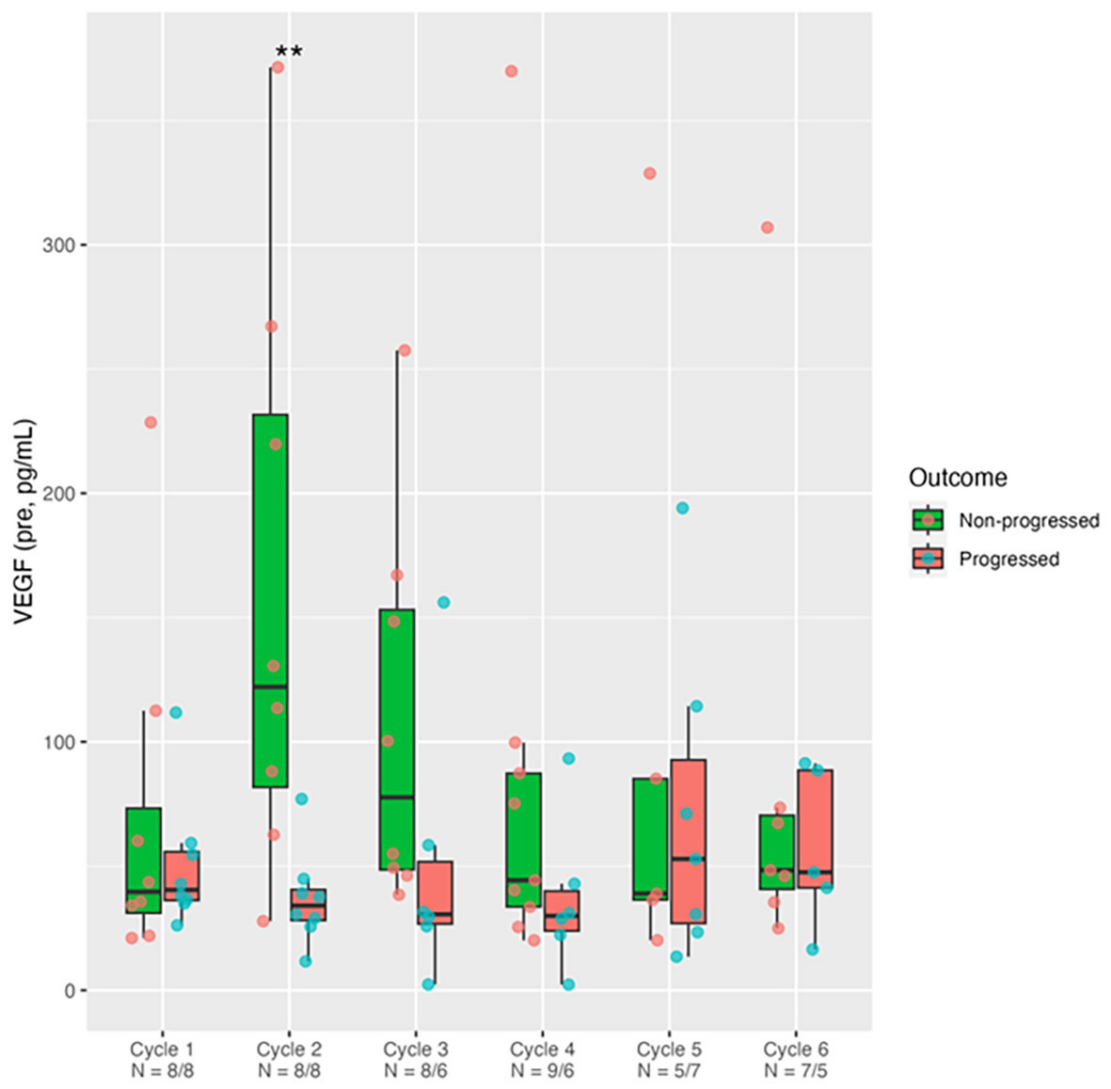
VEGF levels pre-ATRA treatment. Measurement of plasma VEGF by multiplex sandwich immunoassay in PDAC patients just before (Pre) taking ATRA/Gemcitabine/nab-Paclitaxel in the 6 cycles (C1-C6) of STARPAC treatment. Box (median ± interquartile range) and whisker (95% range). VEGF levels in progressed (n=9) vs. non-progressed (n=10) patients, were compared using the Mann-Whitney test. **p <0.01. Samples available (x-axis) for analysis from each cycle/visit varied.

Of all six cytokines tested (IFN-γ, IL-1RA, IL-16, IL-8, RANTES, **Supplementary Figures 1–5**), only VEGF (**Figure 1**) demonstrated a significant increase in the non-progressors compared to patients who progressed at cycle 2 (*p* = 0.007) and persisted at cycle 3 (*p* = 0.081) with subsequent convergence between the two groups in later cycles. The only statistically significant finding (**Table 1**) — VEGF being different between progressors and non-progressors at Cycle 2 — remains significant after Bonferroni correction (*p* = 0.007 < 0.05/6). The median difference between groups at Cycle 2 was 88.02 (95% CI: 32.82–228.84; Mann–Whitney *p* = 0.007). RANTES cytokine (**Supplementary Figure 1**), on the other hand demonstrated some reduction in patients not progressing versus those patients who progressed at Cycle 3 (*p* = 0.054). These measurements were taken before commencing chemotherapy or ATRA. There was no change in plasma cytokine levels before and after giving the drugs (data not shown) as was demonstrated previously for serum PTX3 (6). Whilst CA19–9 decreased in response to chemotherapy, it could not differentiate between patients who progressed and those who did not. Similarly other prognostic variables derived from common laboratory tests such as C-reactive protein-to-albumin ratio (CAR, **Supplementary Figure 7**) and mGPS, (**Supplementary Figure 8**) could not differentiate between patients who progressed and those who did not.

**Table 1:** *P*-values for the significant difference between progressed (n=9) and non-progressed (n=10) patients using Mann-Whitney test (for RANTES, CA19–9 and VEGF) and Fisher’s exact test (for mGPS) (R version 3.5.1).

Potential prognostic variable	Cycle 1	Cycle 2	Cycle 3	Cycle 4	Cycle 5	Cycle 6
Rantes	0.442	0.328	0.054	0.456	0.792	0.073
CA19-9	0.694	0.78	0.888	1	0.758	0.534
VEGF	0.959	**0.007**	0.081	0.224	0.876	1
mGPS	1	0.534	1	1	0.569	1

Statistically significant values highlighted in bold.

## Discussion

VEGF has been known to act as a biomarker in PDAC studies (11). VEGF was also found to be an independent predictor of PDAC when measuring blood, ascites and tumor tissue levels from PDAC patients (12). VEGF has several functions in cancer, including initiating angiogenesis (13), increasing vascular permeability (14) leading to a desmoplastic stroma, and autocrine VEGF signaling in tumor cells contributing to tumorigenesis (15). For example, in lung cancer cells, ATRA treatment increased VEGF-C, VEGF-D (ligands) and VEGFR3 (receptor) expression in a dose-dependent manner (16). Previously, we showed that stromal (stellate cell) modulation could regulate blood vessel density in pancreatic cancer (17). It is possible that the increase in circulating VEGF represents neo-angiogenesis in the PDAC tumor microenvironment (TME) because of stromal (stellate cell) modulation (6), which may lead to reduction in hypoxia and better chemotherapy delivery, both vital obstacles to overcome in treatment of advanced PDAC. Even though VEGF is often associated with progression in other cancers, VEGF elevation in non-progressing patients in our cohort may reflect a tumor that is still relying on structured, VEGF-driven angiogenesis, which is a sign of earlier-stage, more treatable disease (18). Interestingly, despite CA19-9 (and also CAR ratio, mGPS) being unable to differentiate progression status, the VEGF levels by Cycle 2 could distinguish patients who would eventually progress despite treatment.

This hypothesis needs experimental validation to assess if our previous proposal of dual action combination therapy can enhance angiogenesis whilst making chemotherapy more effective (19). Dynamic changes in VEGF levels may serve as a complementary therapy-response biomarker. This could be implemented clinically by sampling plasma at cycles 1 and 2, to identify which patients’ VEGF levels increase upon ATRA treatment.

Although an increase in VEGF levels pre-ATRA treatment in Cycle 2 could indicate that ATRA treatment in Cycle 1 increased VEGF expression, causing angiogenesis, there is also the possibility that VEGF was elevated due to tumor-driven angiogenesis, resulting from hypoxia. Alternatively, the increase in VEGF may be caused by the cytokines and pro-inflammatory signals released during a systemic immune response targeting the tumor area (20).

Whilst this is the first study to evaluate these cytokines in progressors and non-progressors across different treatment cycles, in patients with PDAC on treatment, there are some limitations of this pilot study with a small cohort and no formal power calculation or control arm; thus, limiting its statistical power. The changes demonstrated here should be validated in larger cohort of clinical trial patients, ideally with randomized controlled arms to assess the therapeutic effect of ATRA. This will be addressed and validated by the STARPAC2 trial (https://gtr.ukri.org/projects?ref=MR%2FS036601%2F1) (21). This work also demonstrates that prospectively banked and well-annotated samples from clinical trials, when analyzed retrospectively in a blinded manner could lead to novel biomarker discovery.

## Data Availability

The raw data supporting the conclusions of this article will be made available by the authors, upon reasonable request and without undue reservation.

## Group member of STARPAC Trialists Group

HM Kocher (Chairperson), David J Propper, Fieke EM Froeling, Bristi Basu, Debashis Sarker, Sarah Slater.

## Group member of Barts Pancreas Tissue Bank

Claude Chelala, Archana Ambily, Abu ZM Dayem Ullah, Thomas Dowe, Ahmet Imrali, Rhiannon Roberts, Hemant M Kocher, Christine Hughes.

## Supplementary material

Supplementary Figures 1–6 Measurement of plasma RANTES (SF1), IL-1RA (SF2), IL-8 (SF3), IL-16 (SF4), IFN-γ (SF5) by multiplex sandwich immunoassay and CA19-9 (SF6) by routine biochemistry in PDAC patients before (Pre) taking ATRA in the 6 cycles of ATRA/Gemcitabine/nab-Paclitaxel treatment. Box (median ± interquartile range) and whisker (95% range). Comparisons done for progressed (n=9) vs. non-progressed (n=10) patients, were compared using the Mann-Whitney test. *p <0.05. Samples available (x-axis) for analysis from each cycle varied.

Supplementary Figure 7 C-reactive protein-to-albumin (CAR) ratio was calculated using routine biochemistry values from samples taken before commencement of each cycle of ATRA/Gemcitabine/nab-Paclitaxel treatment. Box (median ± interquartile range) and whisker (95% range). Comparisons done for progressed (n=9) vs. non-progressed (n=10) patients, were compared using the Mann-Whitney test. *p <0.05. Samples available (x-axis) for analysis from each cycle varied.

Supplementary Figure 8 The proportion of mGPS (modified Glasgow Prognostic Score) in progressed (n=9) and non-progressed (n=10) patients at each cycle. mGPS is an inflammation-based prognostic score which combines the serum elevation of CRP (score 0 if < 10mg/L and 1 if ≧̸10 mg/L) and the decrease in albumin concentration (score 1 if 35 mg/L and 0 if albumin ≧̸35 mg/L).
