# Supplementary material for: Plasma vascular endothelial growth factor levels are a potential therapy-response biomarker for pancreatic cancer
Source: Front Oncol. 2026 Jan 9;15:1672385. doi: 10.3389/fonc.2025.1672385 (PMC12827075; doi:10.3389/fonc.2025.1672385)
Supplement: Supplementary file 2 [file DataSheet2.pdf]

RANTES

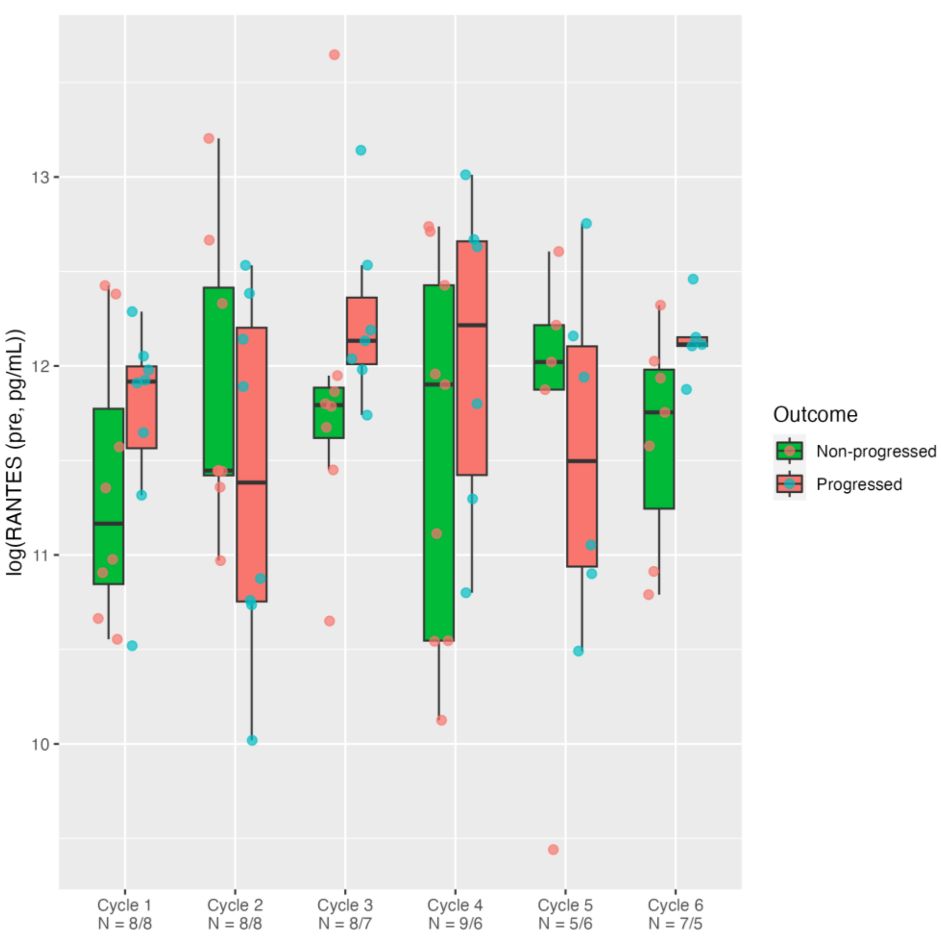

Figure S1

IL-1RA

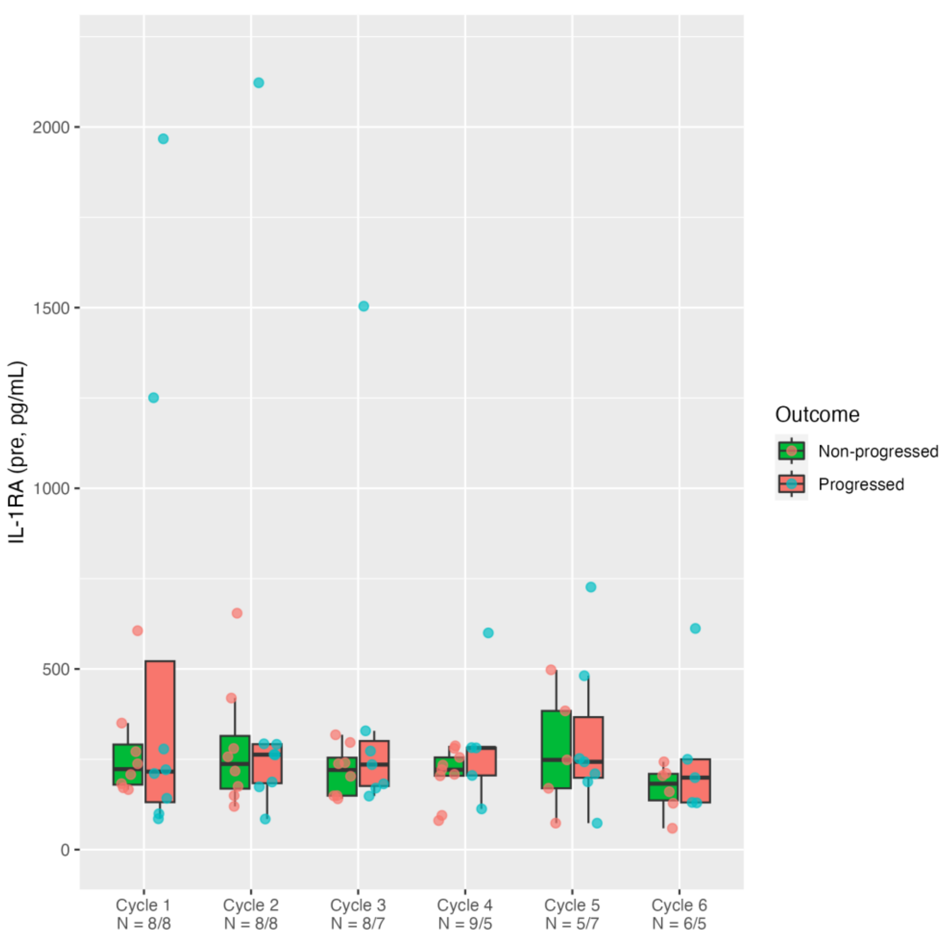

Figure S2

IL-8

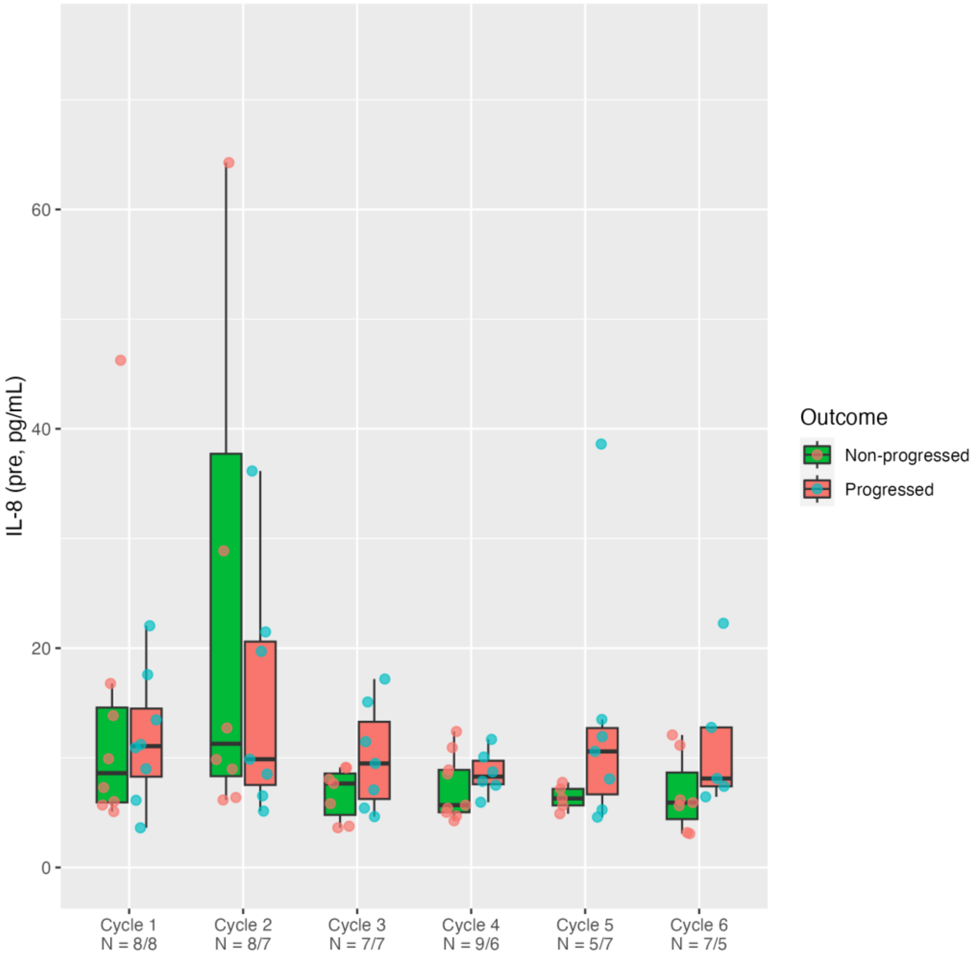

Figure S3

IL-16

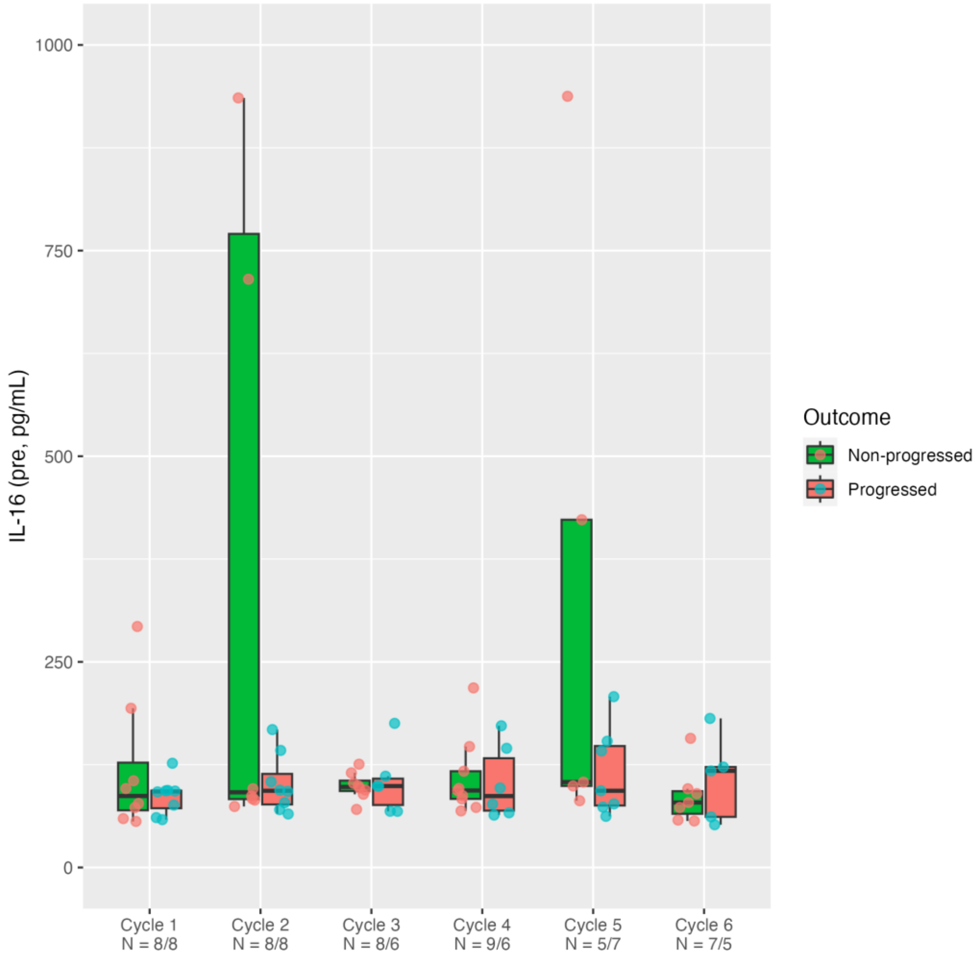

Figure S4

IFN-gamma

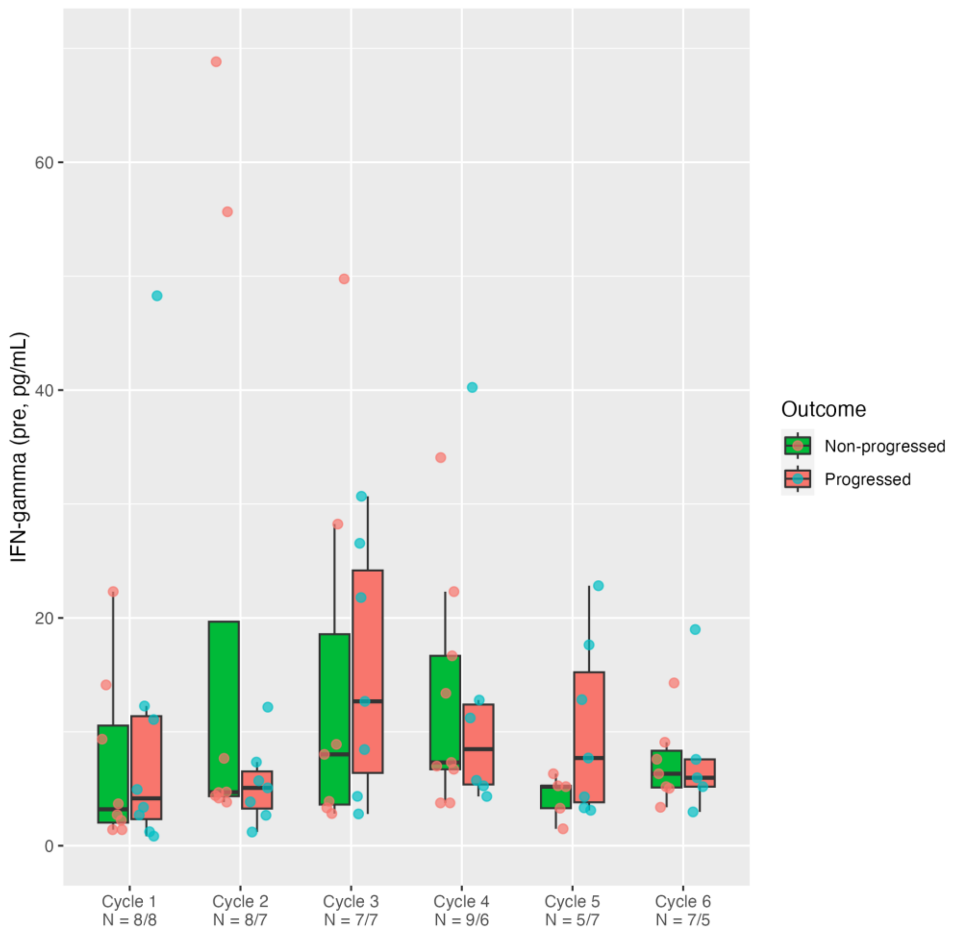

Figure S5

CA19-9

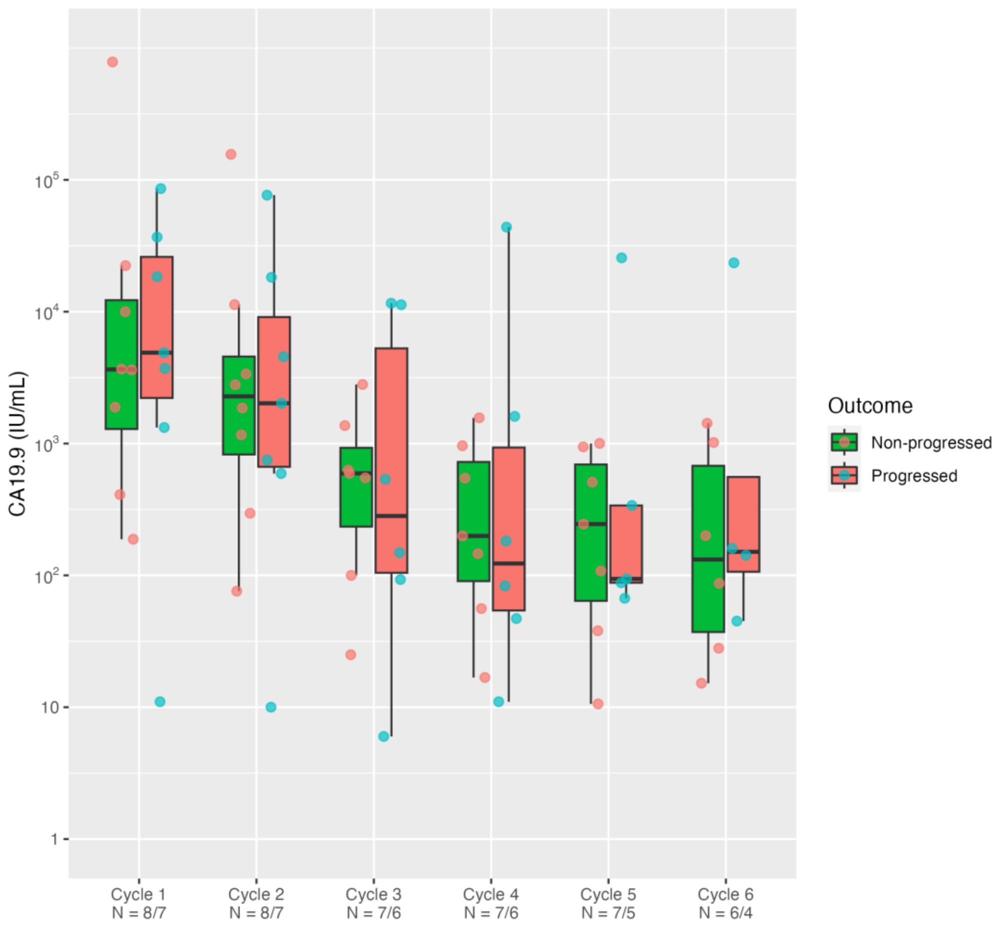

Figure S6

CAR

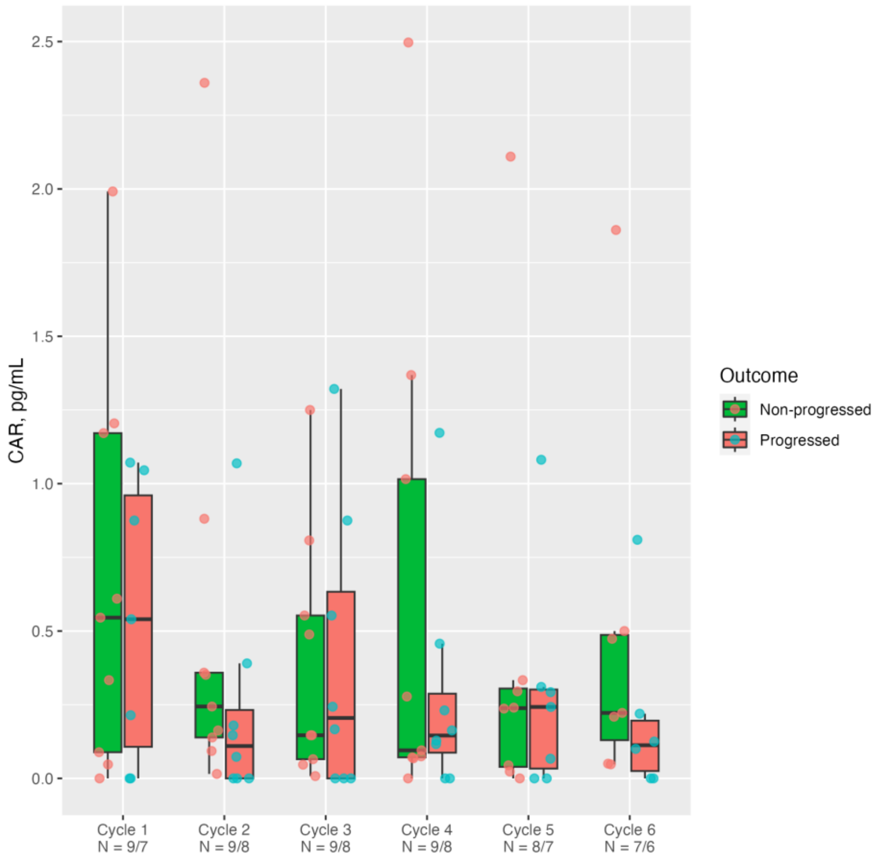

Figure S7

mGPS

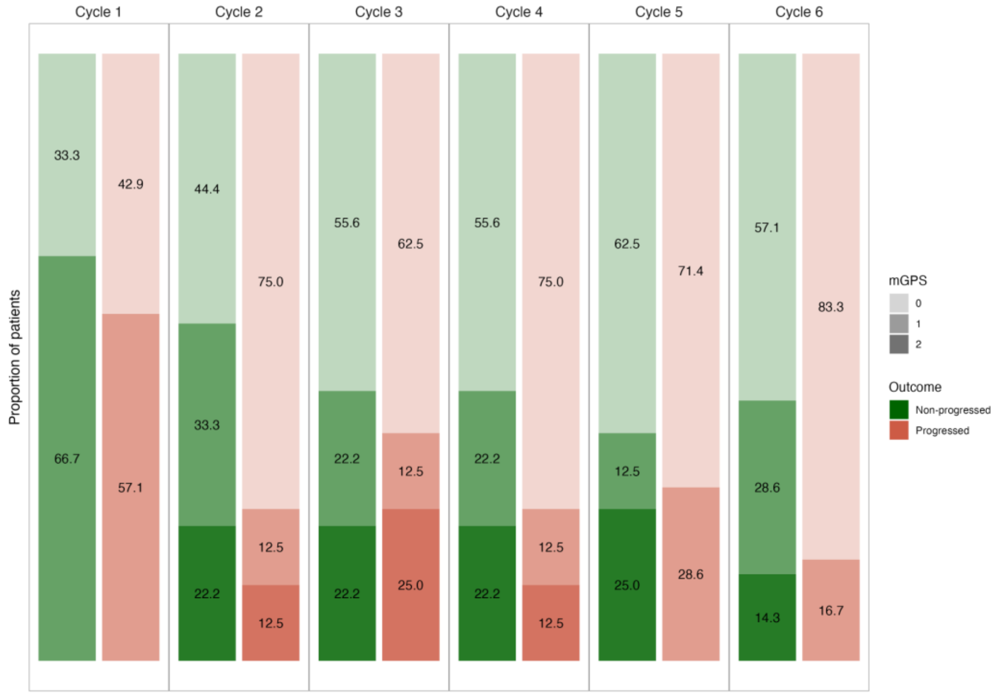

Figure S8
